# Knowledge transfer from macro-world to micro-world: enhancing 3D Cryo-ET classification through fine-tuning video-based deep models

**DOI:** 10.1093/bioinformatics/btae368

**Published:** 2024-06-18

**Authors:** Sabhay Jain, Xingjian Li, Min Xu

**Affiliations:** Electrical Engineering Department, Indian Institute of Technology Kanpur, Kanpur, Uttar Pradesh, 208016, India; Ray and Stephanie Lane Computational Biology Department, Carnegie Mellon University, Pittsburgh, Pennsylvania, 15213, United States; Ray and Stephanie Lane Computational Biology Department, Carnegie Mellon University, Pittsburgh, Pennsylvania, 15213, United States

## Abstract

**Motivation:**

Deep learning models have achieved remarkable success in a wide range of natural-world tasks, such as vision, language, and speech recognition. These accomplishments are largely attributed to the availability of open-source large-scale datasets. More importantly, pre-trained foundational model learnings exhibit a surprising degree of transferability to downstream tasks, enabling efficient learning even with limited training examples. However, the application of such natural-domain models to the domain of tiny Cryo-Electron Tomography (Cryo-ET) images has been a relatively unexplored frontier. This research is motivated by the intuition that 3D Cryo-ET voxel data can be conceptually viewed as a sequence of progressively evolving video frames.

**Results:**

Leveraging the above insight, we propose a novel approach that involves the utilization of 3D models pre-trained on large-scale video datasets to enhance Cryo-ET subtomogram classification. Our experiments, conducted on both simulated and real Cryo-ET datasets, reveal compelling results. The use of video initialization not only demonstrates improvements in classification accuracy but also substantially reduces training costs. Further analyses provide additional evidence of the value of video initialization in enhancing subtomogram feature extraction. Additionally, we observe that video initialization yields similar positive effects when applied to medical 3D classification tasks, underscoring the potential of cross-domain knowledge transfer from video-based models to advance the state-of-the-art in a wide range of biological and medical data types.

**Availability and implementation:**

https://github.com/xulabs/aitom.

## 1 Introduction

Cryo-electron tomography (Cryo-ET) (Gan and Jensen 2012) has emerged as a powerful tool, offering researchers an unprecedented glimpse into the microscopic world of biological particles with remarkable clarity and in near-native conditions. This groundbreaking technique involves the reconstruction of multi-angle projections, resulting in high-resolution 3D tomograms. These tomograms serve as invaluable windows into the intricate structures that underlie biological mechanisms, including the complex interactions and dynamic behaviors of macromolecules (Murata and Wolf 2018). Among the myriad applications of Cryo-ET, this article places a particular emphasis on *subtomogram classification*, a pivotal component for unraveling the mysteries of the entire cellular environment. By honing in on the classification of individual macromolecular structures within the subtomograms, researchers pave the way for a deeper understanding of the fundamental processes governing life at the cellular level.

Achieving reliable subtomogram classification poses a significant challenge, primarily stemming from the scarcity of high-quality labeled data. This challenge can be broken down into several critical aspects. Firstly, the acquisition of Cryo-ET images is cost-prohibitive because of the expensive nature of the required equipment, limiting the availability of large datasets. Secondly, to preserve cellular integrity, the electron dose during imaging must be constrained, leading to lower signal-to-noise ratios (SNRs) in the captured data (Turk and Baumeister 2020). Thirdly, inherent physical constraints of both the instrument and sample thickness result in a phenomenon known as the “missing wedge,” further complicating data acquisition (Kudryashev *et al.* 2012, Lučić *et al.* 2013). Consequently, the scarcity of data increases the risk of overfitting, a classic and ongoing concern in the realm of machine learning. While most existing solutions resort to data simulation (Pei *et al.* 2016, Liu *et al.* 2020a) to augment the training set or few-shot learning algorithms (Yu *et al.* 2021), the complex algorithmic requirements pose challenges for biologists, necessitating extensive design and debugging efforts.

On the other hand, the large-scale natural image datasets like ImageNet (Deng *et al.* 2009) and COCO (Lin *et al.* 2014) have played a pivotal role in the remarkable success of deep learning on diverse computer vision tasks (Krizhevsky *et al.* 2012, He *et al.* 2016). The striking transferability of models well-trained on these expansive general datasets has propelled fine-tuning into a prevalent paradigm, which has also been applied in fields of medicine and biology. Despite these achievements, the practical solutions that have thrived in 2D images have yet to be explored comprehensively for 3D Cryo-ET data. This gap can be attributed to lack of powerful general pre-trained models for 3D vision. To the best of our knowledge, existing studies in the realm of subtomogram classification rely on random initialization, rendering deep learning less reliable and inefficient in the absence of a large-scale dataset. Closing this gap and unlocking the potential of deep learning in Cryo-ET data analysis remain pivotal challenges.

**Figure 1.**
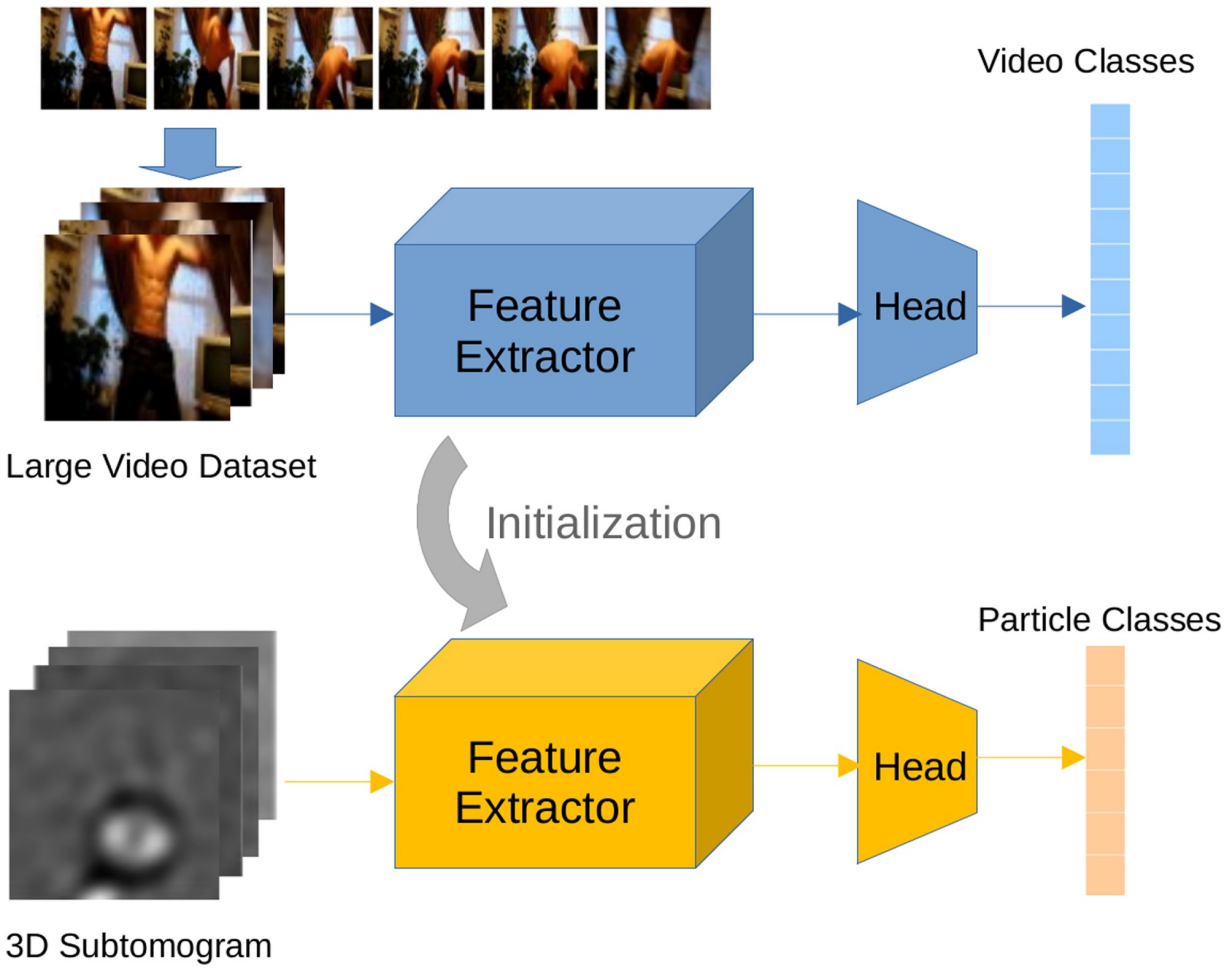
Illustration of subtomogram classification with video-based deep models as initialization. Channel replication is used to adapt 3D video models on single channel subtomogram data. The video sample is from Kinetics-400 (Kay *et al.* 2017).

Motivated by the notable success of the “pre-training and fine-tuning” paradigm within 2D domains, our empirical exploration extends this concept to the realm of 3D subtomogram data. In particular, we leverage pre-trained weights from video data as the initialization for our model. The framework is demonstrated in Fig. 1. The reason of this choice lies in 2-folds. Firstly, it is rational to consider subtomogram slices as sequentially evolving video frames since they share the fundamental attribute of continuity. Secondly, video datasets offer distinct advantages, including low annotation costs, data richness, and fewer privacy concerns when compared to specialized domains such as medical and biological fields.

**Figure 2.**
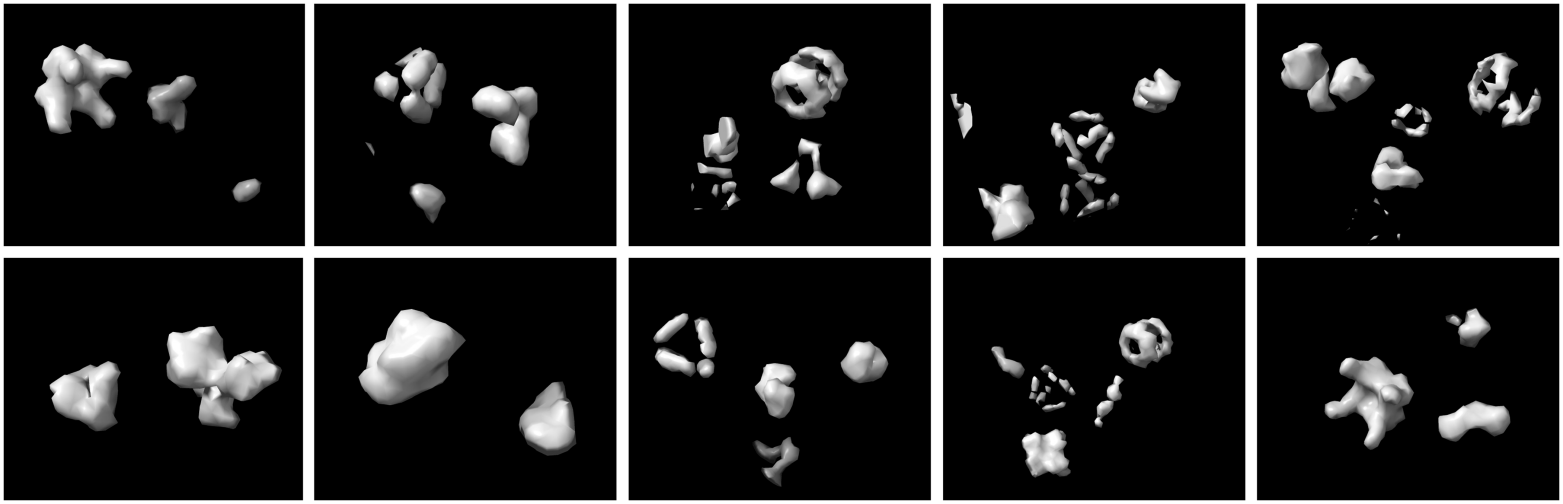
Synthetic structures in the simulated dataset (Liu *et al.* 2020a). The names of the structures from left to right are 1bxn, 1f1b, 1yg6, 2byu, 2h12 in the first row, and 2ldb, 3gl1, 3hhb, 4d4r, 6t3e in the second row.

Our experiments encompassed both simulated (Figure 2) and privately acquired real datasets for subtomogram classification. Leveraging video initialization yielded clear advantages in both performance and efficiency. Notably, it outperformed the baseline (i.e. random initialization) by an impressive 19.19%, 17.67% in absolute accuracy on 5% and 10% of real training dataset, respectively, and 16% in absolute accuracy on 25% of the simulated dataset with 50% reduction in the training efforts. Furthermore, such performance improvements require no additional coding efforts. Our adaptation of the “pre-training and fine-tuning” paradigm opens doors to enhanced insights and efficiency in the analysis of 3D subtomogram data, fostering opportunities for groundbreaking discoveries in various scientific and research endeavors.

## 2 Related work

### 2.1 Subtomogram classification

Identifying particles of interest is a critical step for *in situ* Cryo-ET image analysis. One typical example is the study of virus–host interactions, where viruses interact with host cells in highly specific ways, involving complex molecular machinery and dynamic interactions. In order to understand these interactions and the mechanisms underlying viral infection, it is crucial to accurately identify the viral particles within the cellular context.

Existing techniques such as template matching have been extensively applied for particle recognition. However, their performance is far from satisfactory given the highly noisy images and conformational changes of biological structures (Moebel *et al.* 2021). For example, to construct a 3D tomogram, rotations of the sample axis yield 2D projections from various angles. However, high rotation angles thicken the sample, hindering imaging. Consequently, reconstructed 3D tomograms exhibit missing information in the affected wedge-shaped regions, named the missing wedge effect.

Machine learning-based subtomogram classification allows researchers to categorize and distinguish these variations within the data through more robust feature learning. This classification process not only enhances our understanding of fundamental biological processes, but also facilitates drug discovery and the development of targeted therapies by revealing molecular mechanisms with unprecedented detail.

Deep neural networks (DNNs, Simonyan and Zisserman 2014, He *et al.* 2016) have been successfully applied on subtomogram classification (Che *et al.* 2018, Gupta *et al.* 2022). Further efforts have been made to address the challenge caused by data limitation. Existing work have taken two primary directions. One approach involves data augmentation to expand the labeled dataset. Researchers leverage their understanding of biology to create simulated Cryo-ET tomograms and subtomograms with predefined structures (Pei *et al.* 2016, Liu *et al.* 2020a). The advantage here is that manually designed strategies ensure accuracy of generated labels by exposing all details. However, a notable drawback is the significant feature discrepancy between simulated and real data, limiting its real-world applicability, particularly for novel structures. Additionally, the heavy reliance on existing biological knowledge and predefined rules constrains its versatility. The other prevalent approach aims to enhance learning performance with limited examples, incorporating techniques like few-shot/one-shot learning (Yu *et al.* 2021), active learning (Du *et al.* 2021), and semi-supervised learning (Liu *et al.* 2019b). Inspired by the success of self-supervised learning on natural images (Gupta *et al.* 2022), extends the fashion to subtomogram classification, achieving state-of-the-art (SOTA) accuracy on both simulated and real data.

Although the aforementioned approaches also aim to solve the issue of data scarcity, they are based on additional assumptions, which have limited their applications. For example, few-shot/one-shot learning (Yu *et al.* 2021) and semi-supervised learning (Liu *et al.* 2019b) solutions require a large amount of unlabeled data with a similar distribution to the labeled set. Active learning (Du *et al.* 2021) involves selecting the most informative examples for annotation; however, it assumes that we already have an adapted model to extract accurate features.

### 2.2 Pre-training and fine-tuning

The rational of the fine-tuning paradigm lies in the finding of the impressive transferability of DNNs trained on large-scale general-purpose datasets across a range of downstream tasks (Yosinski *et al.* 2014). Consequently, fine-tuning pre-trained models for adapting to new tasks has gained popularity in real-world applications. To further enhance knowledge transfer, various methods have been explored to optimize the utilization of pre-trained models during fine-tuning. A significant portion of such research adopted a common idea referred to as “shrinking towards chosen parameters,” which aims to overcome the risk of catastrophic forgetting of the general knowledge contained in pre-trained models. Building upon this assumption, several algorithms have been developed, implementing different constraints on model parameters such as *L*^2^-SP (Xuhong *et al.* 2018), DELTA (Li *et al.* 2019), and MARS (Gouk *et al.* 2021). These algorithms show clear benefits especially when the source and target domain are similar, e.g. both are natural images.

Previous research works have also investigated transfer learning, especially the pre-training and fine-tuning paradigm, in biomedical image analysis. Several review papers (Cheplygina 2019, Kim *et al.* 2022, Kora *et al.* 2022) summarized the approaches in recent papers and confirmed the value of deep transfer learning in solving biomedical problems. For example (Hon and Khan 2017), improved the accuracy of Alzheimer’s disease classification from MRI images by utilizing ImageNet pre-trained models. However, previous studies mostly focused on 2D medical images or slices and considered only 2D natural images as the source dataset. For 3D biomedical image analysis, the rich video resources in the natural vision domain have not been exploited before.

### 2.3 Video analysis with deep learning

Deep learning represents a powerful approach to extract meaningful information from dynamic visual data (Karpathy *et al.* 2014, Arnab *et al.* 2021). DNNs tailored for video analysis leverage the temporal dimension inherent in videos to capture motion patterns, temporal dependencies, and spatio–temporal interactions. Similar to the solutions for other spatial and temporal data types, deep models for video analysis typically employ convolutional neural networks (CNNs, Karpathy *et al.* 2014), recurrent neural networks (RNNs, Yang *et al.* 2017), or the combined architectures like Conv-LSTM (Ge *et al.* 2019), to effectively process sequential data. Popular video analysis tasks include action recognition, activity detection, video segmentation, and video captioning and so on. In this article, we will adopt the 3D-CNN (Hara *et al.* 2018) and Video Vision Transformer (ViViT Arnab *et al.* 2021) architecture, and reuse the pre-trained weights learned from large-scale video data to facilitate biological image analysis.

## 3 Method

We use DNNs as the machine learning model in our transfer learning approach. The network consists of a general-purpose feature extractor *f* and a classification head *g*. In deep transfer learning, *f* aims to learn data features with a task-agnostic architecture. *g* is responsible for the final prediction based on deep features provided by *f*. We denote the whole model by z=g°f parameterized with ω=(θ,ϕ), where *θ* and ϕ are associated with *f* and *g*, respectively.

For both pre-training and fine-tuning, we adopt the principle of Structural Risk Minimization (SRM) to train the model over labeled training dataset D={(x,y)}. Specifically, we will minimize the cross entropy loss between predicted probabilities and ground truth as

(1) $$L(\omega )=\frac{1}{\left|D\right|}\sum_{(x,y)\in D}L_{\text{ce}}(z(x;\omega ),y)+\lambda \Omega (\omega ),$$

where Ω is a regularizer to prevent over-fitting, and *λ* is used to balance the effect of empirical risk minimization and model complexity restriction. We adopt standard weight decay to realize the regularizer, i.e. $\Omega (\omega )=||\omega |{|}^{2}$.

To enhance robustness of deep learning, we adopt stochastic gradient descent (SGD) to minimize the learning objective of Equation (1). Therefore, the learnable parameter *ω* will be iteratively updated on a random batch of examples at each step t=1,2,…,T as

(2) $$\omega_{t}=\omega_{t-1}-\eta \frac{\partial L_{B}(\omega_{t-1})}{\partial \omega_{t-1}},$$

where *η* refers to the learning rate.

To making use of general vision features from natural videos, the network *z* is first trained over large-scale datasets. It is worth noting that we don’t need to repeat this process due to the availability of open-source pre-trained models. Denote *θ^S^* as the parameter of the pre-trained feature extractor *f*. In the fine-tuning stage, we initialize the target network with $\theta_{0}=\theta^{S}$ and $\phi_{0}$ with random initialization. Then the whole parameter *ω* will be solved according to Equation (2). Based on the above transfer learning framework, we will introduce our specific choices of source/target datasets and model architectures in the following section.

## 4 Experimental setting

Our approach primarily revolves around fine-tuning several 3D deep learning models pre-trained on the large-scale video dataset (video initialization), such as Kinetics-400 (video action classification dataset Kay *et al.* 2017), for subtomogram classification and other 3D biomedical image classification tasks.

### 4.1 Pre-trained dataset and models

#### 4.1.1 Kinetics-400 dataset

It is a large-scale, high-quality dataset of video clips covering 400 human action classes. These videos encompass a wide array of human activities, spanning from interactions between humans and objects, such as playing musical instruments, to interpersonal human-to-human interactions, including gestures like handshakes and hugs. We leverage a range of models pre-trained on this dataset for tasks like subtomogram and MedMNIST3D classification.

#### 4.1.2 3D ResNets for action classification

The adoption of 3D convolution has become increasingly popular with the inception of large-scale video datasets, as it can capture spatio-temporal features. Notably, the introduction of the 3D ResNet architecture, as proposed in Hara *et al.* (2017), has showcased remarkable accuracy in the Kinetics-400 action classification task. In our research, we leveraged a pre-trained 3D ResNet-34 model as a fundamental component in our experiments.

#### 4.1.3 Video vision transformer

Vision transformer models have recently achieved SOTA results for various computer vision tasks. The ViViT proposed in Arnab *et al.* (2021) uses a pure-transformer-based approach to extract the spatio-temporal features from the input video. For our research, we employed the pre-trained “google/vivit-b-16x2-kinetics400” ViViT model as a foundational element in our experimentation. Specifically, it adopted the 12-layer basic ViViT Factorised Encoder proposed in the article and used 16 × 16 × 2 as the input patch size for Transformers. The model was trained over the Kinetics 400 dataset for 30 epochs as suggested by the article.

### 4.2 Target datasets

#### 4.2.1 Simulated Cyro-ET data

Numerous approaches are available for simulating Cryo-ET data. In this study, we adopt the framework developed by Liu *et al.* (2019b). Their method employs an efficient gradient descent-based technique to generate 3D Cryo-ET subtomogram images of a target macromolecule situated in a crowded environment with randomly positioned neighboring macromolecules. The macromolecules undergo random rotations and translations. Furthermore, the simulation process incorporates tomographic artifacts, such as the missing wedge effect and electron optical factors, to emulate experimentally acquired Cryo-ET images.

In the experiments, we followed previous practice (Gupta *et al.* 2022) to select 10 simulated structures including 1bxn, 1f1b, 1yg6, 2byu, 2h12, 2ldb, 3gl1, 3hhb, 4d4r, and 6t3e. We used simulated data with an SNR of 0.03 for our experimentation. The dataset consists of 10 classes with 500 samples per class, and each subtomogram is of size 32^3^ (32 × 32 × 32). The 5000 samples are divided into a 60:20:20 ratio for training, validation, and test split.

#### 4.2.2 Real Cryo-ET data

The real-world dataset utilized in this study was derived from the Noble single-particle dataset (Noble *et al.* 2018). We used the approach outlined in Gupta et al. (2022). In this method, potential structural regions were extracted from each tomogram within the Noble single-particle dataset employing the Difference-of-Gaussians (DoG) method. The particle structures are rabbit muscle aldolase, hemagglutinin, T20S proteasome, DNAB helicase–helicase, glutamate dehydrogenase, insulin-bound insulin receptor, and apoferritin. Subsequently, the top 1000 sub-volumes were chosen based on cross-correlation scores, and for each class, a manual selection process was undertaken to pick 400 subtomograms.

The final real-world dataset comprises seven classes, each consisting of 400 samples. Each subtomogram has a size of 28^3^ (28 × 28× 28). To facilitate model training and evaluation, the 2800 samples are partitioned in a 3:1:1 ratio for training, validation, and testing, for comparison with Gupta *et al.* (2022) and 1:1 ratio for training and testing for comparison with Liu *et al.* (2020b).

#### 4.2.3 MedMNIST3D

We use MedMNIST3D as an additional dataset, which is a large-scale MNIST like collection of standardized 3D biomedical images. This dataset encompasses six distinct collections, namely OrganMNIST3D, NoduleMNIST3D, AdrenalMNIST3D, FractureMNIST3D, VesselMNIST3D, and SynapseMNIST3D. All images are pre-processed into 28^3^ (28 × 28× 28) with the corresponding classification labels.

### 4.3 Deep learning strategies

This part describes the strategies for training our target datasets, i.e. subtomograms and other 3D biomedical images.

#### 4.3.1 Data pre-processing and augmentation

We perform pre-processing to ensure the shape of our Cryo-ET data fits the video pre-trained models. Specifically, each subtomogram is resized into the shape of (32 × 128 × 128) for consistency with video clips. The new pixels in the last two dimensions are generated by interpolation. For data augmentation, we follow the approach presented in Gupta *et al.* (2022), which comprises two main steps. First, a random resized crop is taken with a 50% probability, where the initial image is scaled between 0.5 and 1. Second, a random affine transformation is applied with a 50% probability, involving rotation within −45 to 45° along the *z*-axis, horizontal translation up to 10% of the image’s width, vertical translation up to 10% of the image’s height, and potential scaling by a factor up to 10%.

Note that the 3D subtomogram data have three symmetric spatial axes, which is different from video data. Therefore, it does not matter which axis acts as the temporal dimension when reusing the video model. In our implementation, we use the first axis of subtomogram to fit the temporal axis in the video model and treat the remaining two as the spatial axes.

#### 4.3.2 Optimization

When employing our video initialization strategy, we train the model with a batch size of 32 for 25% and 100% of dataset and 16 for 5% and 10% of dataset over 30 epochs, with a learning rate starting from 1e-4 and delaying it by 0.1 after 15 and 22 epochs. To ensure sufficient adaptation from the video domain to the Cryo-ET data domain, all the model parameters are updated during the fine-tuning process. For the random initialization, we undertake an extended training regimen spanning 100 epochs. In this scenario, the learning rate begins at 1e-3 and is scheduled for reduction by 0.1 after 50 and 75 epochs. All the models are trained using the categorical cross-entropy loss and we used Accuracy and AUC (Area under the ROC Curve) as the evaluation metrics.

### 4.4 Hardware and software environments

All of our experiments are conducted on a single NVIDIA GeForce RTX 3090 GPU card. The version of Nvidia driver is 535.161.07 and CUDA is 12.2. We use Python 3.8 and Pytorch 1.10 on a Ubuntu 22.04 system.

## 5 Main findings

### 5.1 Results of feature extraction

In deep transfer learning, the quality of the pre-trained features is a key factor to estimate the potential value of transfer learning. There are both theoretical and practical evidence in previous studies. For example, Liu *et al.* (2019a) reveals that good pre-trained weights provide a flatter initial loss surface for the target task. Another direction of work (Gouk *et al.* 2021, Li and Zhang 2021) indicates that an initial model with relevant features is helpful to constrain the upper bound of the generalization error on the target task. In complex AI systems which consist of both DNNs and traditional handcrafted features (Lai and Deng 2018), the features extracted from pre-trained models are directly applied in the system without fine-tuning the feature extractor. In those applications, the quality of pre-trained embeddings is critical to the performance of the overall system.

**Figure 3.**
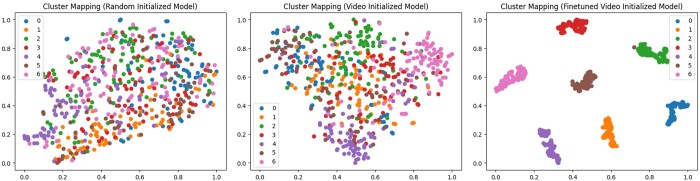
2D visualization of the embeddings generated using random initialization (left), video initialization (middle), and the fine-tuned model using 3D-ResNet-34 as the architecture. The categories represent 1bxn, 1f1b, 1yg6, 2byu, 2h12, 2ldb, and 3gl1, respectively. Noisy structures are generated to simulate real environments. It can be seen that the video-initialized model has similar subtomograms clustered together.

To evaluate whether pre-trained video models provide better initialization for subtomograms, we perform a preliminary experiment on feature extraction. Specifically, we feed subtomograms into the fixed pre-trained video model and extract the deep embedding for each subtomogram. Random examples from seven classes are selected in this experiment. The deep embeddings are then projected onto a 2D space for visualization using t-SNE (Van der Maaten and Hinton 2008). t-SNE is a dimensionality reduction technique commonly used for visualizing high-dimensional data in lower-dimensional spaces, particularly effective in revealing clusters and patterns in complex data. It aims to preserve the local and global structure of the data by modeling pairwise similarities between data points in the high-dimensional space and mapping them to a lower-dimensional space. The 2D visualization results are shown in Fig. 3, where each color refers to a class. It can be seen that the video pre-trained model has similar subtomogram samples clustered together, i.e. the points belonging to the same category occur closely in the 2D space. We also show the results of our fine-tuned model as a reference. As seen in Fig. 3, after fine-tuning, the samples from different classes are separated clearly.

### 5.2 Results of fine-tuning

**Figure 4.**
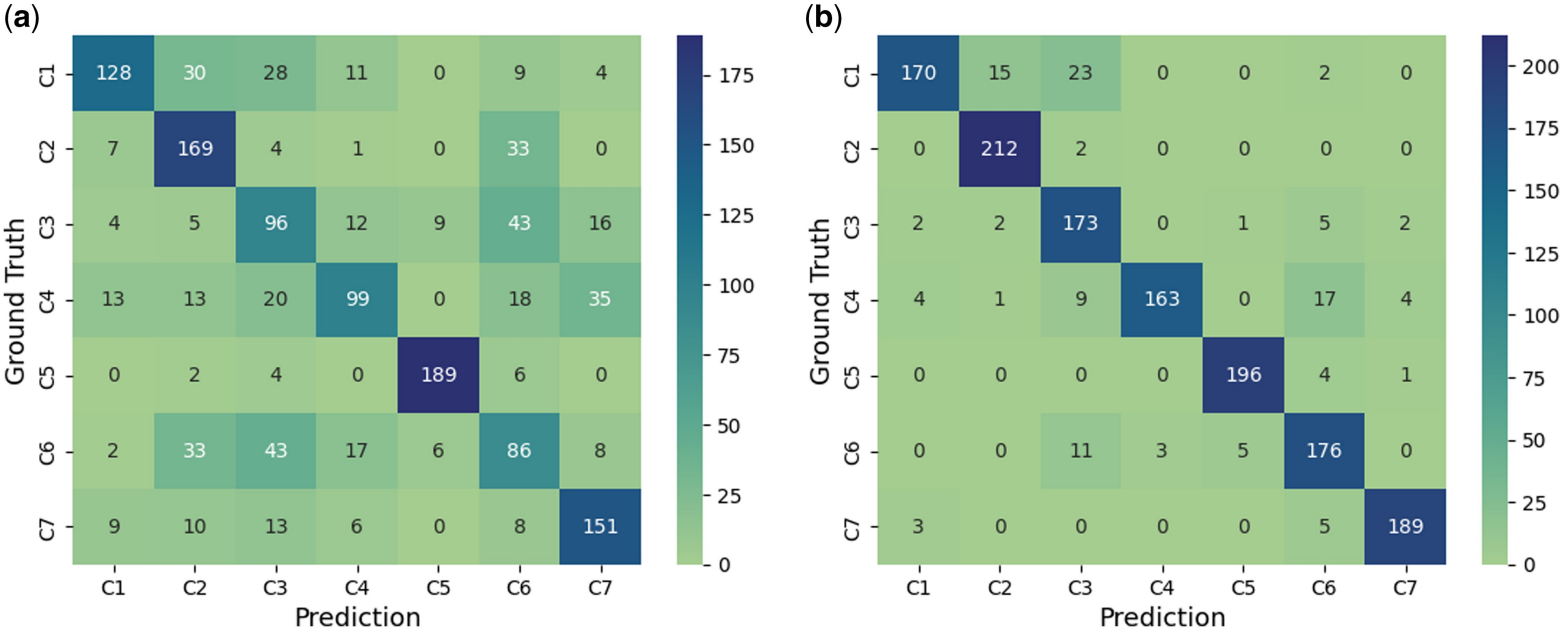
Confusion matrix for models trained on 5% real data. The seven classes are C1 = rabbit muscle aldolase, C2 = hemagglutinin, C3 = T20S proteasome, C4 = DNAB helicase-helicase, C5 = glutamate dehydrogenase, C6 = insulin-bound insulin receptor, and C7 = apoferritin. (a) Random initialization. (b) Video initialization.

We further conducted a series of experiments aimed at fine-tuning the 3D-ResNet-34 model for subtomogram classification using both random and video initialization approaches. We randomly sampled 25% and 100% of the training size for the simulated dataset and 5%, 10%, 25%, and 100% of the training size for real-world data. Each experiment was repeated five times to capture variations in performance, and we reported the average accuracy and AUC (Area under the ROC Curve). The results of our investigations are presented in Table 1. (Note that the SSP paper Gupta *et al.* 2022 aims to design self-supervised learning to improve DNN training, which is different from our objective. The SSP code may not adopt the optimal hyperparameters in model training, leading to poor performance. We reported the performance of SSP as a reference, showing that our paper achieved the SOTA performance in terms of subtomogram classification.) We observed that the video initialization approach outperforms random initialization for both simulated and real Cryo-ET data. This difference in accuracy becomes even more pronounced when we use limited data (5%, 10% for Real Data and 25% for Simulated Data) for training the model. When more training data, the performance difference decreases. This can be mostly attributed to the nature of catastrophic forgetting in the context of DNNs (French 1999, Chen *et al.* 2019). Using the video initialization approach, we achieved the highest accuracy of 87.38% on the simulated data and 99.57% on the real data. Through the confusion matrix presented in Fig. 4, we observed that on the most challenging 5% real data as the training set, our video initialization method delivered a much more accurate discrimination between complex structures including DNAB helicase-helicase (C4) and insulin-bound insulin receptor (C6) compared to random initialization. Additionally, we conducted experiments on 3D biomedical datasets from MedMNIST3D, as shown in Table 2. Notably, our video initialization approach demonstrated remarkable performance improvements, particularly for the SynapseMNIST3D, VesselMNIST3D and OrganMNIST3D datasets.

**Table 1.** Comparison of subtomogram classification accuracy (%) and AUC on real and simulated Cryo-ET data.^a^

Dataset	(%) labelled	Initialization	Accuracy	AUC
Simulated data	25	Random	65.88	0.9237
	25	Ours	**81.88** (+16.00)	**0.9733**
	25	SSP (Gupta *et al.* 2022)	31.4	
	100	Random	85.34	0.9885
	100	Ours	**87.38** (+2.04)	**0.9905**
	100	SSP (Gupta *et al.* 2022)	58.8	
Real data	5	Random	68.71	0.91173
	5	Ours	**87.90** (+19.19)	**0.982**
	5	Semi-supervised (Liu *et al.* 2019b)	78.21	
	10	Random	77.55	0.95792
	10	Ours	**95.22** (+17.67)	**0.9976**
	10	Semi-supervised (Liu *et al.* 2019b)	84.64	
	25	Random	90.78	0.9923
	25	Ours	**98.5** (+7.72)	0.**9989**
	25	SSP (Gupta *et al.* 2022)	98.4	
	100	Random	98.67	0.9998
	100	Ours	**99.57** (+0.9)	**0.9999**
	100	SSP (Gupta *et al.* 2022)	98.5	

a Classifier (3D-ResNet-34) with Video initialization performs much better than classifier with random initialized weights. Gupta *et al.* (2022) presents the results of best self-supervised pretraining (SSP) strategy using the RB3D model, and Liu *et al.* (2019b) presents semi-supervised approach using 3D Autoencoding Classifier.

Bold value refers to the best one among each experimental group.

**Table 2.** Comparison of classification accuracy (%) and AUC on MedMNIST3D datasets.

Dataset	Initialization	Accuracy	AUC
OrganMNIST3D	Random	90.26	0.9937
	Ours	**96.52**	**0.9988** (+0.0051)
SynapseMNIST3D	Random	82.89	0.8674
	Ours	**90.45**	**0.9352** (+0.0678)
VesselMNIST3D	Random	**94.34**	0.917
	Ours	94.03	**0.9509** (+0.0339)
NoduleMNIST3D	Random	86.25	0.8786
	Ours	**87.16**	**0.883** (+0.0044)
AdrenalMNIST3D	Random	82.88	0.8615
	Ours	**83.28**	**0.8634** (+0.0019)
FractureMNIST3D	Random	**52.83**	0.696
	Ours	51	**0.6991** (+0.0031)

Bold value refers to the best one among each experimental group.

**Figure 5.**
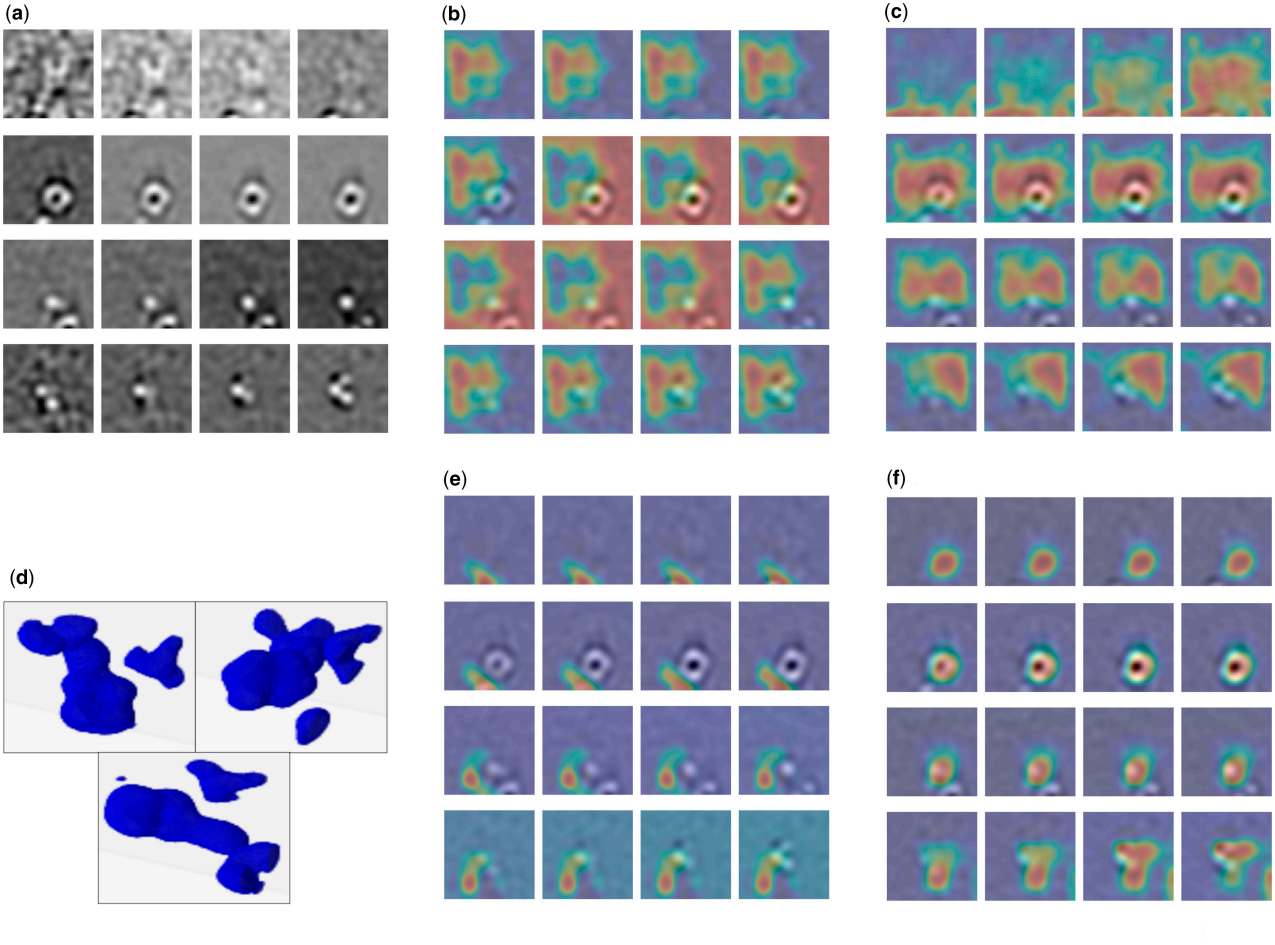
Grad-Cam visualization of layer3 of 3D-ResNet-34. Video initialization captures the subtomogram region with greater accuracy in comparison to the randomly initialized model. (a) Cryo-ET subtomogram sample. (b) Random initialization. (c) Video initialization. (d) 3D density map of the sample. (e) Fine-tuning baseline. (f) Fine-tuning (ours).

**Figure 6.**
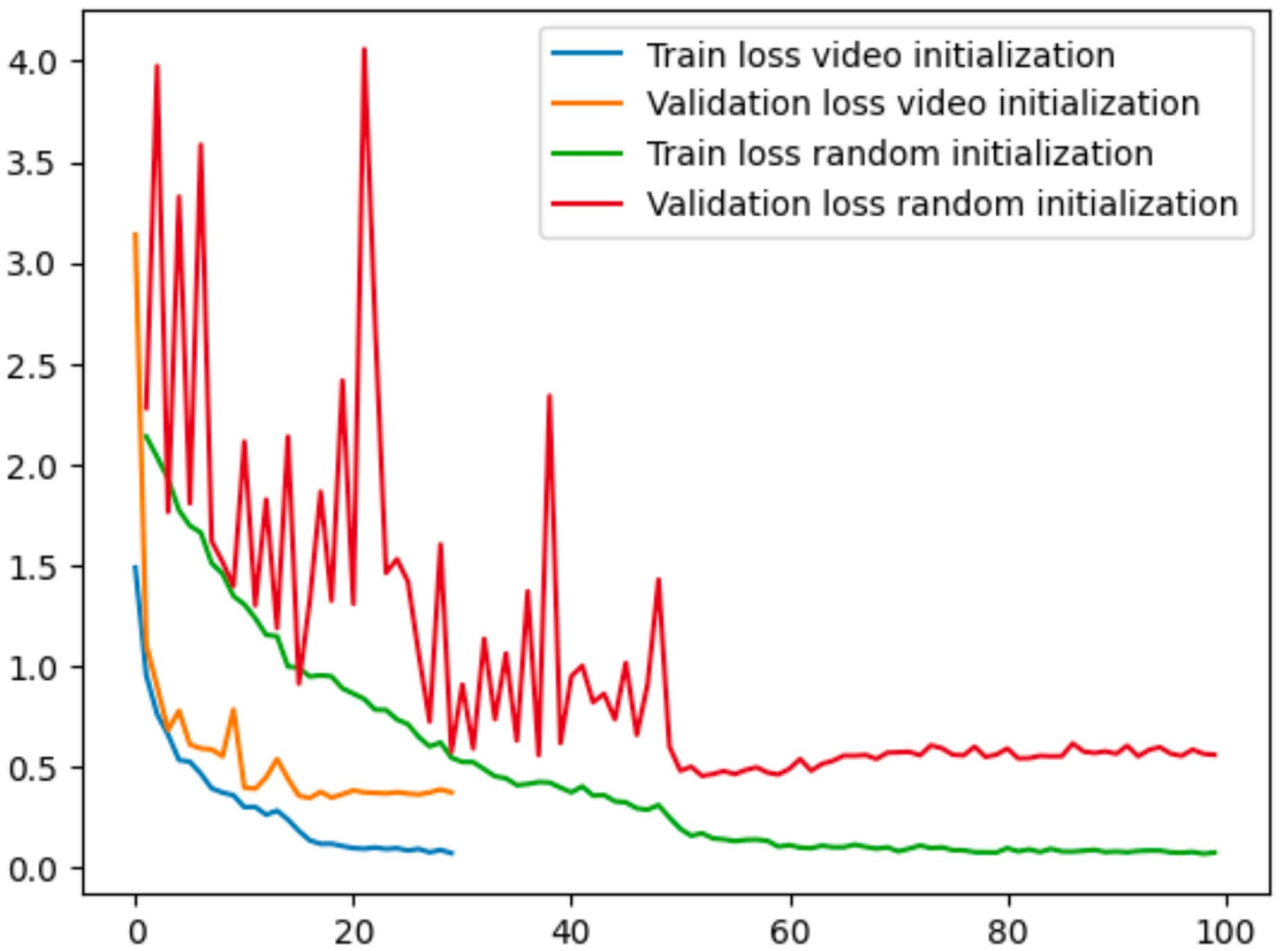
Train and validation loss curves for Video initialization and Random initialization.

Furthermore, in Fig. 5, we present the Grad-CAM visualizations (Selvaraju *et al.* 2017) for a sample subtomogram image, roughly illustrating the regions crucial for classification decisions. These visualizations were generated using M3d-CAM (Gotkowski *et al.* 2021). Evidently, the model fine-tuned with video initialization captures the subtomogram region with significantly greater accuracy in comparison to the randomly initialized model. A similar phenomenon can be discovered when comparing the video initialized model (without fine-tuning) with a random initialized model. compared to random initialization. These observations confirm that our model does not only deliver more accurate predictions, its decision logic is also more reliable. We additionally chart the training and validation loss curves (Fig. 6) during the training of the 3D-ResNet-34 model on simulated Cryo-ET data. Clearly, the video initialization approach exhibits significantly faster convergence, requiring fewer epochs than the random initialization.

We also conducted experiments applying our video initialization technique to ViViT. More specifically, we utilized a pre-trained ViViT model, which had been trained on the Kinetics-400 dataset (“google/vivit-b-16x2-kinetics400” Arnab *et al.* 2021), as the basis for video initialization. Our approach achieved an overall accuracy of 86.6% on 100% Simulated Cryo-ET test data. In contrast, when we attempted to train a model with random initialization, we faced challenges due to limited data availability (only 3000 samples for training) and scanty compute resources (only able to train the model with batch size 1 due to GPU memory constraints), resulting in a significantly lower test accuracy of 32.3%.

## 6 Conclusion

This article introduces an innovative example of cross-domain transfer learning, facilitating knowledge transfer from the extensive domain of large-scale video datasets to the highly specialized domains of microbiology and biomedicine. Our findings reveal that, in general, the video initialization approach exhibits superior performance and higher efficiency, and the difference becomes much more significant when we have fewer training samples. Thus, reusing the spatio-temporal features learned from extensive video domains can be a practical approach for deep learning on microbiological and biomedical domains.
